# Engaging Community Health Centers to understand their perceptions and interest in longitudinal cohort research on diabetes mellitus in Native Hawaiian communities: Initial insights from the Waimānalo community

**DOI:** 10.3389/fpubh.2022.1035600

**Published:** 2022-12-08

**Authors:** Marjorie K. Leimomi Mala Mau, Nicole Kau'i Baumhofer Merritt, Kamuela Werner, Mary Frances Oneha

**Affiliations:** ^1^Department of Native Hawaiian Health, John A. Burns School of Medicine, University of Hawai'i at Mānoa, Honolulu, HI, United States; ^2^Division of Mathematics, Natural and Health Sciences, University of Hawai'i - West O'ahu, Kapole, HI, United States; ^3^Waimānalo Health Center, Waimānalo, HI, United States

**Keywords:** community-based participatory research (CBPR), Native Hawaiian, Pacific Islander, cohort study perceptions, qualitative research, diabetes mellitus

## Abstract

**Introduction:**

Despite decades of research on diabetes mellitus (DM) and other health disparities affecting Native Hawaiian and Pacific Islander (NHPI) populations, little is known about the disease mechanisms that underlie these health disparities. Ideally, a longitudinal cohort study is one of the best research design tools to examine underlying mechanisms of disease in health disparity conditions such as DM. The study purpose is to understand the perspectives and insights of people (*n* = 29) living in NHPI communities about conducting longitudinal cohort studies aimed at understanding mechanisms of health disparities in NHPI populations.

**Methods:**

All interviews were audio-recorded, transcribed and de-identified into written transcripts for thematic content analysis.

**Results:**

Four major themes emerged: 1) Diabetes and other **health disparities is a community priority** because these diseases touch nearly everyone; 2) Cohort-type research and its outcomes should extend beyond data collection to include **data sharing using a cultural context approach**; 3) Cohort-type research can **directly benefit everyone, especially youth, through education** on new, locally-derived knowledge; 4) A longterm benefit of cohort-type research should be to support **“generational change” in the community**.

**Discussion:**

In summary, potential “cohort-type research” (a.k.a. longitudinal cohort study designs) was perceived as a worthy endeavor because health disparities, such as DM, affects nearly everyone in the community. Cohort-type research is important to NHPI communities as it holds promise for impacting “generational change” on health and wellbeing through the sharing of new community-derived knowledge.

## Introduction

Type 2 diabetes mellitus (DM) remains a major health disparities problem among Native Hawaiians, Pacific Islander People (NHPI), and racial/ethnic minority populations in the USA (1, 2). To confront the health burden of DM and health disparities in Hawai'i, especially among the NHPI population, several community organizations joined the Ulu Network beginning in 2003 and partnered with academic-based researchers from the Center for Native and Pacific Health Disparities Research (CNPHDR) at the University of Hawai‘i at Mānoa, John A. Burns School of Medicine's Department of Native Hawaiian Health to work together with a common goal of reversing health disparities prevalent among the Center's priority populations, including NHPIs.

**Figure 1:**
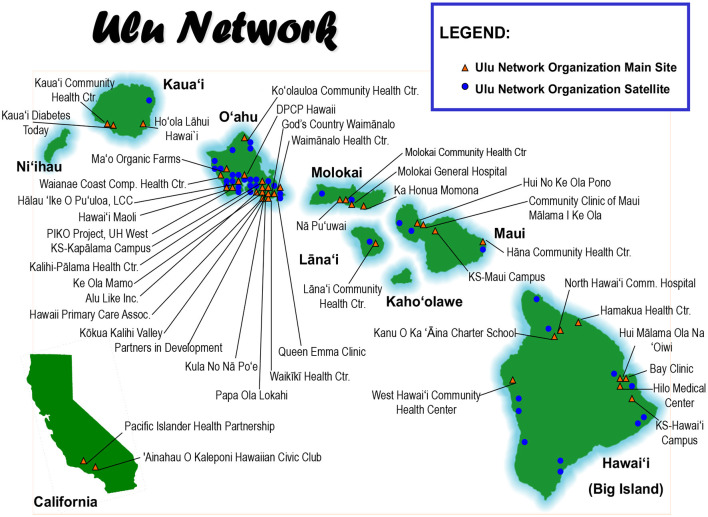
Center for Native and Pacific Health Disparities Research's map of the Ulu network community-based organizations in the State of Hawai'i and Southern California.

The Ulu Network is a voluntary coalition of 35+ organizations who have partnered with the CNPHDR to reduce health disparities in ~70+ locations throughout the State of Hawai'i and Southern California (Figure 1). Extensive collaborations between Ulu Network and the CNPHDR includes: >50 community-directed health education and training workshops, 12+ peer-led health education interventions implemented in the communities they serve. The community-engaged activities included knowledge exchange (e.g., community as part of the scientific team), technical assistance (e.g., data collection, etc.), shared resources (conference sponsorship, program materials, food models, etc.) and actual program funding (i.e., Ulu dissemination awards, etc.). Over the years, these bi-directional, community-led projects have been an overwhelming success to build capacity across the Ulu Network members and the empirically-tested programs have been used by trained community-peers with remarkable fidelity and consistency resulting in positive clinical improvements and successful skill-building of community-based peer-educators (3–7). Today this relationship remains a vibrant, synergistic partnership that has enabled the development of new collaborations both within and external to the CNPHDR, across other academic units at the University of Hawai'i and have also sparked other community-to-community collaborations (5).

Yet, despite the years of successful community engaged research, training and dissemination programs, a number of Ulu Network organizations continued to struggle with the growing burden of DM in their communities, especially with the rising prevalence of obesity and DM in youth (2, 8–10). Indeed, recent national studies showing a decline in DM incidence in the USA highlights the growing gap between most minority populations and whites in the USA. Moreover, multiple studies have shown that DM onset and complications continue to occur at younger ages (10–15 years younger) compared with their white counterparts (11–14). Thus, the health inequity gap continues to widen despite health benefits realized by primarily USA whites.

In September 2016, all 14 Federally Qualified Health Centers (FQHCs) of the Ulu Network were invited to a meeting to specifically discuss the future plans of how best to address diabetes disparities that were occurring in Ulu Network communities. The idea of developing a new longitudinal, observational cohort study of NHPIs with community-led organizations was openly discussed and debated. The overall purpose for creating a new “first of its kind” longitudinal cohort of NHPIs was to enhance our capacity to increase understanding of mechanism of disease, investigate risk exposures and resilience factors in NHPIs prior to onset of DM and associated complications which would, in turn, inform future interventions or programs. Many of the FQHC representatives raised important questions about how such studies were desperately needed and how it would impact their communities and clinics. Some FQHC representatives expressed concern that research studies without direct health programs to benefit communities would be challenging and less favorable. While other FQHC representatives were willing to explore the possibilities of developing a research resource, such as a “prospective, longitudinal observational cohort study,” to better understand the underlying cause of common health disparities in their community. All attendees were aware of the tremendous effort it would take to develop, establish and sustain a longitudinal cohort study drawn from NHPI communities and the expenditure of time and funding. After ~3 months of ongoing dialog and communications with Ulu Network FQHCs, a single FQHC, Waimānalo Health Center (WHC), remained willing and able to take next steps to actually collect data from their community members to gain a deeper understanding of what the Waimānalo community thought about longitudinal “cohort-type” research focused on NHPI health disparities such as DM.

The purpose of this study was to assess the perspectives and recommendations of a single NHPI community served by the WHC, on the potential for conducting longitudinal, observational cohort designed research with NHPI people to address health disparities, such as DM, in their community.

## Methods

### Study setting

This research study was conducted in the community of Waimānalo, located on the east side of the island of O‘ahu, in the State of Hawai‘i. This is a close-knit, rural community with stunning natural resources from the mountain (*mauka*) to the ocean (*makai*) that make farming, fishing, canoe paddling and other ‘*āina* (land)-based activities the foundation of Waimānalo's economy and lifestyle (15, 16). The community of Waimānalo values its connection to the ‘*āina*, the preservation of agricultural lands, and the perpetuation of Native Hawaiian cultural practices (15).

Waimānalo is a clearly defined geographic community with a population of 6,278 (16). The population demographics reflects a median age of 34.2 years, 15% of individuals are below the poverty level, 3% are unemployed, and 4% are uninsured (16). Thirty percent (30.2%) of the residents in this community are Native Hawaiian and/or Pacific Islander ancestry (16).

This study was co-designed by academic-based researchers (MKM, NKBM, KW) of the CNPHDR and WHC clinical leadership (MFO) and audio recorded and officially transcribed into a redacted written format.

### Participants

Purposive sampling was used to recruit participants from the larger Waimānalo community using email, written invitation, or personal contact. Key informants were recruited from attendees at the WHC's established cultural classes and from leadership at Waimānalo community organizations, including the Waimānalo Neighborhood Board and Hawaiian Homestead Association. Focus group participants were recruited from attendees at the WHC's established cultural and diabetes prevention/self-management program classes. Open recruitment from interested community participants who heard about the study, but were not WHC class attendees, were also invited to participate in one of the scheduled focus groups. Key informant interviewees were not eligible for participation in the focus groups and vice a versa. Eligibility criteria included (a) age 18 years or older, (b) resident of the Waimānalo community or a member of a faith-based, health-based, or grassroots community organization located in Waimānalo. Participation in the focus groups and key informant interviews were not limited to only NHPI. Although the intention of the study was to explore the idea of a longitudinal cohort study to address DM in NHPI, the community that the cohort study would be situated in, is comprised of a diverse mix of racial and ethnic groups. Non-NHPI individuals included in the study have deep and longstanding ties to the Waimānalo community through their years of participation in community-based organizations.

### Study procedures

Focus group and key informant interviews were conducted by WHC staff and administrative leadership according to protocol using a single prepared moderator's guide (see Supplementary material). All interviews were audio recorded and transcribed. The transcripts were independently coded and then collaboratively reviewed to determine consensus by two academic-based (MKM, NKBM), one “hybrid” academic-community-based (KW) and two community-based members (MFO, CSH) of the research team. Thematic analysis was conducted through a common template in which the reviewing community-based researcher identified key words or phrases related to specific questions asked of participants, then identified the theme related to those responses (17). All participants gave written informed consent prior to any data collection. This study was submitted for IRB approval and deemed exempt by the University of Hawai'i (UH) Committee on Human Subjects.

### Moderator guide

The introduction to interview questions included a basic description of the different common types of research study designs (i.e., observational (e.g., non-interventional), clinical trials (e.g., testing an intervention, etc.) with a specific focus on a longitudinal, prospective, observational study design we referred to as “cohort-type” research. We provided brief descriptions in plain-language about how data is generally collected in longitudinal, cohort-type research and what kinds of data are often collected including biospecimens preserved for future analyses. Participants were generally engaged and enthusiastic about the idea of “cohort-type” research and asked questions to the moderator or interviewer to better understand the study process and then proceded with the moderator guide questions. According to protocol, focus groups and informant interviews were initiated using a cultural “talk story” approach to stimulate conversation and to establish a comfortable, “safe” place in which participants could share their opinions and provide feedback without recourse (See Appendix: Description of a “Cohort Study” and Moderator Guide) (18).

### Analysis

Written transcript of the audio recorded interviews (focus group and informant) were initially reviewed independently by the research team members and then collated by group discussion, using inductive and deductive approaches to reach group consensus. Initially, all members of the research team reviewed the transcripts to familiarize themselves with the data. During the first round of coding, research team members identified codes that were categorized by the broad themes explored in the interview guide topics: understanding of research designs, culturally-appropriate handling of biospecimens, community engagement, and thoughts surrounding a community/academic partnership. Following the first round of coding, the research team met to discuss, define, and refine themes. A second round of coding identified key words and phrases reflecting the refined themes. We performed synthesized group member-checking to enhance trustworthiness of the data ~6–9 months following data collection. Participants were invited to share their opinions on the summarized data presented and their reflections are incorporated into the final results.

## Results

Of the 29 participants who volunteered for the study six participated as key informant interviewees and 23 participated in five focus groups. Most were women (72%), NHPI (83%) and age >55 years old (59%) (Table 1). Most (83%) of the participants were long time residents of the Waimānalo community (>15 years) with more than half (52%) of the participants had lived in Waimānalo for >30 years. Nearly three-fourths of the participants (71%) lived in multi-generational households and more than half (55%) lived with 3–4 generations residing in the same home. There were a few notable differences between the key informant and focus group participant demographics. All of the key informants were NHPI, one-third (33%) were 25 to 45 years old, and none were extreme-longtime (>50 years) residents of Waimānalo. Whereas, in the focus groups, a substantial percentage (78%) were NHPI, less of the focus group were 25 to 45 years old (22%) and nearly one-third (30%) were extreme-longtime (>50 years) residents of Waimānalo.

**Table 1:** Participant characteristics (*N* = 29).

**Characteristic**	**Key informants** ***n* = 6 (%)**	**Focus group** ***n* = 23 (%)**	**Total** ***N* = 29 (%)**
**Age (years)**			
25–45	2 (33)	5 (22)	7 (24)
46–55	—	5 (22)	5 (17)
56–70	3 (50)	12 (52)	15 (52)
>70	1 (17)	1 (4)	2 (7)
**Gender**			
Female	3 (50)	18 (78)	21 (72)
Male	2 (33)	5 (22)	7 (24)
Trans-Female	1 (17)	—	1 (3)
**Race (self-reported)^a^**			
Native Hawaiian, Pacific Islander	6 (100)	18 (78)	24 (83)
White	—	4 (17)	4 (14)
Asian	—	1 (4)	1 (3)
**Marital status^b^**			
Married	2 (33)	11 (48)	13 (45)
Not married	4 (67)	12 (52)	16 (55)
**Years living in Waimānalo community**			
≤15 Yrs	—	5 (22)	5 (17)
16–30 Yrs	2 (33)	7 (30)	9 (31)
31–50 Yrs	4 (67)	4 (17)	8 (28)
More than 50 Yrs	—	7 (30)	7 (24)
**Number of generations in household^c^**			
One	2 (33)	6 (26)	8 (28)
Two	1 (17)	3 (13)	4 (14)
Three	3 (50)	7 (30)	10 (34)
Four	—	6 (26)	6 (21)

a NHPI, Native Hawaiian, Pacific Islander.

b Not married = single, divorce/separate, widow, etc.

c Total responses = 28 due to one missing response.

Four major themes and nine sub-themes (Table 2) emerged and are summarized as:

**Table 2:** Summary of Waimānalo Community's perspectives on ”Cohort-type“ Research aimed at Diabetes Health Disparities in Native Hawaiian and Pacific Islander Populations.

**Themes / sub-themes**	**Participant quotes**
**1. Diabetes health disparities touches the lives of nearly all participants**	
Diabetes longstanding impact on multiple generations and race/ethnic groups	[A] cohort study [that] concentrate[s] on diabetes... would be a great benefit...because... it runs pretty deep in not only Pacific Islanders, but Asians and Native Hawaiians... I can speak from personal experience that it exists on my paternal side., so... any research related to this disease and how it could... potentially alter our childrens [risk for diabetes is good]
New and improved diabetes information is needed to be effective	... it would only be effective if the right information was given out.... There needs to be new information, new ways, new awareness
**2. Research needs to extend beyond observational data collection to include cultural activities, values and build upon community resources**	
Recommendations for data sharing	I would really suggest against just mailing things to people. If there is some kind of interaction between the cohort, that would make it real personal. That would make it more exciting, just engaging relationships
Community approaches to data collection	Food would be a culturally appropriate method of collecting data because it draws them in...
	If there's an educational aspect or actual hands on something or other. If it's just data collection, sometimes it might be harder to have the participants keep coming
**3. Direct benefits of research in the community should include education to families and youth**	
Sharing new knowledge with youth	…Even in the schools, why not talk to them about... [diabetes], because they experience it through their families. They see family [members] die from it, lose limbs, and they don't understand it. Why can't they teach them that in school?... you need to take care your health, you know this is important
Healthy nutrition resources (access, cooking, etc.) at school translate to home environment	...For the children of Waimanalo, access to food is important, and if the schools can be a breeding ground for [learning about] healthy eating, it potentially could translate at home...
	Substitute like ‘ulu (breadfruit) or kalo (taro) for potato and have those types of cooking demonstrations. Make food more innovative, different approach to see how food is utilized instead of the normal beef stew, is there something we can use instead of beef or make it just all vegetables; instead of using potatoes, you have kalo, …
Sharing new knowledge within cultural context	Using “olelo Hawai'i or even just ma ka hana ka “ike (in working, one learns), having that interaction and seeing it happen, just knowing
**4. Research should support “generational change” that communities can implement themselves**	
Investment in the health of future generations	... it's for the future generations, so really,... you have to think about your kids, think about your grand kids, you know what I mean? Think about the kids in the community because when you talk about kids, that's important. That's like our basis of living, we all work to support our kids
	I hope that they are able to be changed, have a different type of eating pattern or eating behaviors from previous generations as we have known the different health disparities that we are facing because of whatever foods or even environment that affects us
	I would like to see the [diabetes] spiral stop. It continues, and I see that it is really difficult to change... Especially in community, because of what's around you. I have tried, I have really tried to change just small little changes, you know, brown rice for white rice at our agency and the pushback is just so amazing
Self-sustaining health that communities can do for themselves	Self-sustainability is important to... health, so learning more about growing [our] own food and [our] own medicine. I know my goal for our community is to become self-sustainable with healthy food and understand more about our herbal medicine which is my goal for my family too

(1) **Diabetes health disparities touches the lives of nearly all participants**. *Need to learn more about DM and especially “new” information and “new ways” of improving DM care*.

Participants recognized that diabetes is a “wide spread problem” in their community. Cohort-type research could be a tool to learn more about how to prevent and address diabetes in an impactful way. Nearly all participants shared personal experiences related to living with DM in their own lives, their parents, grandparents, and/or their childrens lives. While participants were eager to know more about DM to affect the next generation, they yearned for “new” information and “new” ways of addressing DM. Educating youth on this new information gathered through this “new” approach to research could be carried out in many places such as schools, churches, or at home.

(2) **Research needs to extend beyond the observational data collection to include cultural activities, values and build upon community resources**. *Research should include data sharing as well as data collection*.

Participants felt that any research in their community needed to go beyond data observations only to include cultural and traditional activities such as storytelling and relationship building opportunities with researchers to educate the community about how and why research is done and to share new findings. Further, opportunities to build upon existing community resources, such as schools, churches, senior housing, neighborhood boards, social clubs, and other healthcare resources, would enhance participation and retention.

(3) **Direct benefits of research in the community should include education to families and youth**. “*Cohort-type” research should directly benefit the community through sharing of new knowledge with everyone... especially youth*.

Community participants felt that initial and sustained community participation would require efforts to influence behavior changes, keep participants motivated, and provide “direct benefit” to the community. The type of activities needed to meet this expectation might be considered “atypical” for cohort studies in the past and examples included education to families and especially youth through schools, hands-on experiences, and new information that can be applied within the community to improve and prevent DM. Outreach to and collaboration with individuals, community leaders, and key community groups about education were recommended as a means to gain sustained support.

Participants were especially enthusiastic about the possibility of a different kind of cohort-type research that could be more culturally appropriate, based on relationships, and be inclusive of community training and the integration of cultural values and practices. Examples shared included the use of ‘*Olelo Hawai‘i* (Hawaiian language), *mo‘olelo* (storytelling) to communicate research findings, and perhaps cooking demonstrations or planting and harvesting of homegrown foods.

(4) **Research should support “generational change” that communities can implement for themselves**. *Support “generational change.”*

A cohort-type study should use its research findings and information to support community-led “generational change” that they could implement themselves. The participants wanted to see this future generational change translated into “useable forms of information” to promote healthy eating habits, affordable foods and growing their own food to become self-sustainable to make it affordable for everyone. Participants expressed that breaking-up of old patterns of inter-generational eating that is unhealthful, is critical to self-sustainability and a thriving community.

## Discussion

This initial study enabled us to explore the opinions, perspectives and insights of a single NHPI community on the idea of research that, by design, is observational in nature and aimed at understanding disease mechanisms of DM, as identified by the community as an important health disparity. We learned that the community participants understood the scientific importance of a “cohort-type” study and were enthusiastic about conducting this type of research because it offered the possibility of creating new information and approaches for a serious disease in their community. Yet, the “how” of implementing this type of research within NHPI communities, such as theirs, was viewed as equally important to its success as much as the science itself. Much of the subsequent discussion focused on potential recommendations to help create and sustain a potential “cohort-type study” with a focus on their own community. Waimānalo community participants recommended that any new knowledge gained from research to elucidate underlying mechanisms of DM risk and health disparities among NHPIs, be shared first with everyone in the community, especially youth. Information should be conveyed in plain language to educate and remind the community organizations of its vital part in supporting the research results. The need for NHPI-specific data that would improve their risk for reversing current DM trends was noted as a high priority. Concern for sustaining the “cohort-type” study long enough for it to provide new information and possible breakthrough discoveries was an important concern. Recommendations on sustainability from the Waimānalo community included integration of community resources, cultural practices and values in recruitment and retention efforts as key factors for maintaining community participation in the research. This holistic approach of how the community perceived the potential for cohort-type research was also expressed in their hope for the findings of this type of research to benefit future generations of people in their community and to promote positive generational change to reverse health disparities, including DM, in the future. Thus, the emphasis on educating, especially youth, from the community about how best to reverse DM trends by using new information and discoveries produced by cohort-type studies on DM disparities especially in community environments like their own.

In summary, we were encouraged to learn that despite known historical and cultural trauma invoked by research studies performed on NHPI communities in the past, the overriding theme expressed by the participants of this study was refreshingly insightful and encouraging. We learned that longitudinal, “cohort-type research“ was viewed as valuable to understanding and gaining new knowledge about DM in NHPIs. What we learned is that the Waimānalo community considered longitudinal cohort research as something valuable enough to provide the research team with recommendations on the process of implementing a cohort study. They proposed a relatively “new” idea about ”cohort-type research“ studies actually serving a dual purpose. First purpose acknowledged by the NHPI community members would be scientific discovery of a well-designed, longitudinal, prospective cohort study aimed at elucidating underlying mechanisms of DM disparities in NHPIs and other high risk populations. Secondly, that although “cohort-type” studies do not typically provide community outreach and dissemination programs, a potentially “new model” of longitudinal cohort studies designed to serve a dual role of knowledge sharing with the “targeted popuation” as part of the ongoing retention activities. Our study suggests that this type of “knowledge sharing” could take the form of educating youth in schools about DM and how the research being done within the Waimānalo community was contributing to growing ”new research“ discoveries.

Our results are consistent with prior studies conducted in other understudied, health disparate minority populations for whom participation in longitudinal cohort studies have been challenging and poorly delineated (19, 20). Herring et al. (20) conducted focus groups among Black Seventh-Day Adventist church members and found similar barriers to longitudinal cohort studies including lack of any intervention programs or sharing of study results or information to the community. Our study contributes to the existing literature by confirming similar issues in another understudied, health disparate population (i.e., Native Hawaiians and Pacific Islanders). However, in contrast to prior studies, this project was undertaken within a context of a pre-existing longstanding relationship and with the foresight and intent of understanding the communities' genuine concerns and preferences on observational longitudinal cohort research prior to any grant funding. Indeed, much of the existing literature describes challenges and “lessons learned” after funding has already occurred and when enrollment of minority populations may have fallen short (21, 22). We were intentional in our approach for this study by approaching all 14 Ulu Network CHCs to consider the concept of a longitudinal cohort study prior to any funding as the means for sharing of information and building trustworthy relationships. While this study did not explore the reasons for why the other CHCs declined participation, we expect to share our initial findings to the other CHCs to determine their perceptions about longitudinal cohort research. Thus, our study is a first step and a demonstration of how the context of longstanding trustful relationships are the foundation to community engaged research that seeks inclusion of health disparate, understudied populations such as NH and PI and other marginalized populations in the USA.

Of note, we also recognize limitations of our study, including that our results are from a single NHPI community and thus may not be generalizable to other communities at similar risk. We also acknowledge the limited participation of males (*n* = 7, 24% of total) and individuals between 25 and 45 years of age (24% of total) which suggests caution in generalizing our results across genders and younger (<45 years old) age sub-groups. However, we are encouraged by the initial results from the WHC community which is largely NHPI and rural and often mistrustful of research in general. In this study, the participants were diverse and remarkably open to the potential for new types of research studies in their community. In fact, at the end of the formative study, the academic-based researchers were invited back to the WHC and attended two community gatherings to share the results, prior to, manuscript submission (i.e., member checking). The academic-based researchers remain committed to continuing the open discussion about how communities can support research and community-engaged scientists to uncover new discoveries, i.e., underlying mechanisms of DM, a common, persistent and in some cases devasting disease in this and other high risk communities. Our team as a whole felt reasurred that at least for this community, education to youth was a key output of the research and we anticipate engaging other Ulu Network members to learn about their perspectives on the value and suggestions for establishing and sustaining a non-interventional, longitudinal cohort study aimed at health inequities, such as DM, in NHPI communities.

As we move forward with this effort, we intend to build upon our longstanding relationship with Ulu Network members and to specifically invite health care providers such as the FQHCs as they serve as a safety net for any participants who may need medical services for conditions uncovered during the course of research. Inclusion of other indigenous scientists with expertise in genomics, epigenomics and observational epidemiology areas of science may also provide additional insight on “native-driven” models of prospective longitudinal study design. In the end, we anticipate that it will be our longstanding relationship with our NHPI communities (i.e., Ulu Network) which have been built on longstanding trust as the foundation to create a new paradigm for the potential of the first, longitudinal, prospective study on DM risk and health disparities in NHPI communities to be co-led by community and academic leaders as equal stakeholders.

## Author's note

The content is solely the responsibility of the authors and does not represent the views of the National Institutes of Health.

## Data availability statement

The raw data supporting the conclusions of this article will be made available by the authors, upon request and approval.

## Ethics statement

The studies involving human participants were reviewed and approved by the University of Hawai'i Committee on Human Subjects. The patients/participants provided their written informed consent to participate in this study.

## Author contributions

All authors listed have made a substantial, direct, and intellectual contribution to the work and approved it for publication.
