# Erratum: Engaging Community Health Centers to understand their perceptions and interest in longitudinal cohort research on diabetes mellitus in Native Hawaiian communities: Initial insights from the Waimānalo community

**DOI:** 10.3389/fpubh.2023.1143145

**Published:** 2023-01-25

**Authors:** Frontiers Production Office

**Affiliations:** Frontiers Media SA, Lausanne, Switzerland

**Keywords:** community-based participatory research (CBPR), Native Hawaiian, Pacific Islander, cohort study perceptions, qualitative research, diabetes mellitus

Due to a production error, the last author's name was incorrectly written as “Mary Frances Oneha Oneha.” Their surname was duplicated. The correct name is “Mary Frances Oneha.”

The publisher apologizes for this mistake. The original version of this article has been updated.

