# Supplementary material for: Engaging Community Health Centers to understand their perceptions and interest in longitudinal cohort research on diabetes mellitus in Native Hawaiian communities: Initial insights from the Waimānalo community
Source: Front Public Health. 2022 Dec 8;10:1035600. doi: 10.3389/fpubh.2022.1035600 (PMC9780047; doi:10.3389/fpubh.2022.1035600)
Supplement: Supplementary file 1 [file Data_Sheet_1.docx]

**Appendix: Description of a "Cohort Study" and the Moderator Guide for Focus Groups and Informant Interviews**

The moderator guide included a brief description of what a "cohort study" entailed and what a potential participant would be expected to complete, if enrolled. The description of a "cohort study" was as follows: "A cohort study is designed to look at the experiences of persons without disease at time of enrollment and then to follow the person over time (i.e. several years). Enrolling in a cohort study may not benefit the participant immediately. But may benefit your children or grandchildren in the future with new research findings. If you enroll in a cohort study, you will usually be asked to respond to several questionnaires and will be asked to provide a blood sample or other biological samples (i.e. saliva, urine, etc.). Then once a year or every two years, participants will be asked questions about their lived experience on areas such as diet, exercise, stress as well as any new changes to their medical history. This type of monitoring and repeated data collection may occur over several years or decades." Participants were then allowed to reflect and ask any questions prior to introducing the moderator guide questions.

Seven moderator guide questions were posed to participants: (1) *Tell us about your dreams for your children and grandchildren.* (2) *How much value would a potential cohort-type research project bring to this community?* (3) *What would engage community members to participate?* (4) *What would be helpful for young adults to participate?* (5) *How long could this project go-on-for, will participants stay engaged?* (6) *What do you think about the School of Medicine partnering with community organizations to administer this project?* (7) *Is there anything else you would like to add?*
